# The triglyceride glucose-body mass index predicts adverse reproductive outcomes in women with polycystic ovary syndrome undergoing frozen embryo transfer

**DOI:** 10.3389/fendo.2025.1629837

**Published:** 2025-07-30

**Authors:** Ziyin Ding, Kai Liao, Kun Liang, Shuo Zhang, Zhenle Pei, Feifei Zhang, Liming Zhou, Congjian Xu

**Affiliations:** ^1^ Center for Reproductive Medicine, Women and Children’s Hospital of Ningbo University, Ningbo, China; ^2^ Department of Obstetrics and Gynecology, Obstetrics and Gynecology Hospital, Fudan University, Shanghai, China; ^3^ Shanghai Key Laboratory of Female Reproductive Endocrine Related Diseases, Obstetrics and Gynecology Hospital, Fudan University, Shanghai, China; ^4^ The Central Laboratory of Birth Defects Prevention and Control, Women and Children’s Hospital of Ningbo University, Ningbo, China; ^5^ Center for Reproductive Medicine, Ren Ji Hospital, School of Medicine, Shanghai Jiao Tong University, Shanghai, China; ^6^ Department of Obstetrics and Gynecology, Shanghai Medical School, Fudan University, Shanghai, China

**Keywords:** triglyceride glucose-body mass index, polycystic ovary syndrome, adverse reproductive outcomes, miscarriage, frozen embryo transfer

## Abstract

**Objective:**

To investigate the associations between triglyceride glucose-body mass index (TyG-BMI) and reproductive outcomes in women with polycystic ovary syndrome (PCOS) undergoing frozen embryo transfer (FET).

**Methods:**

This retrospective cohort study included PCOS women undergoing their first *in vitro* fertilization (IVF) or intracytoplasmic sperm injection (ICSI) cycle followed by FET from January 2018 to January 2024 at a single reproductive medicine center. Patients were categorized into four groups according to the quartiles of TyG-BMI. Multivariable logistic regression, restricted cubic splines (RCS) and stratified analyses were used to evaluate the associations between TyG-BMI and reproductive outcomes. LASSO regression was performed to identify predictors for miscarriage and receiver operating characteristic (ROC) curve analysis was used to evaluate the predictive power.

**Results:**

A total of 744 women were included in the analysis. After adjusting for covariates, TyG-BMI showed a negative correlation with live birth rate and positive correlations with the risks of miscarriage and gestational diabetes mellitus (GDM) (all P trend < 0.05). RCS models demonstrated linear relationships of TyG-BMI with miscarriage rate, GDM risk and large for gestational age risk (P-overall < 0.05, P-nonlinear > 0.05). These associations remained consistent across all subgroups of the population (all P-interaction > 0.05). ROC analysis revealed that TyG-BMI was predictive of miscarriage (area under the curve (AUC) = 0.627, P < 0.001) with a cutoff value of 180.4. Combined with other identified risk factors, including basal luteinizing hormone, basal follicle stimulating hormone, total cholesterol, testosterone, infertility type and controlled ovarian stimulation protocols, the AUC value increased (AUC = 0.667, P < 0.001) and this model showed good miscarriage prediction performance in most subgroups (AUC > 0.650, P < 0.05), especially in patients with normal or low weight (BMI < 24 kg/m^2^, AUC = 0.743, P < 0.001).

**Conclusion:**

Higher TyG-BMI levels are independently associated with an increased risk of adverse reproductive outcomes in women with PCOS undergoing FET. Additionally, TyG-BMI proves to be a cost-effective tool for the early identification of high-risk groups among PCOS patients, enabling personalized interventions prior to IVF to optimize reproductive outcomes in this population.

## 1 Introduction

Polycystic Ovary Syndrome (PCOS) is one of the most common endocrine disorders in reproductive-aged women, with a global prevalence of approximately 5-18% (depending on the population studied and the diagnostic criteria used) (1). It is characterized by ovulatory dysfunction, hyperandrogenism, and polycystic ovarian morphology, with a significant impact on both reproductive and metabolic health (2). Notably, PCOS accounts for up to 70% of anovulatory infertility cases requiring assisted reproductive technologies (ART), such as *in vitro* fertilization (IVF) (3). Beyond its effects on fertility, PCOS predisposes women to gestational complications, including hypertensive disorders and gestational diabetes mellitus (GDM), while also exerting long-term impacts on offspring health, such as low birth weight (LBW), large for gestational age (LGA), neonatal hypoglycemia, and congenital anomalies (4–8).

Though the exact pathophysiology of PCOS remains elusive, emerging evidence indicates that metabolic disorders, particularly insulin resistance (IR), obesity and dyslipidemia, are critical drivers of PCOS pathogenesis and progression (9). IR and compensatory hyperinsulinemia are prevalent in approximately 75% of PCOS patients (10). These conditions exacerbate hyperandrogenemia by stimulating the overproduction of androgen in ovarian theca cells and inhibiting the synthesis of hepatic sex hormone-binding globulin, perpetuating anovulation and hirsutism (11). Obesity, affecting about 50% of PCOS women across populations, exacerbating IR severity and functional ovarian hyperandrogenism, as well as induces ovarian inflammation and reduces oocyte quality (12, 13). Dyslipidemia, prevalent in up to 70% of cases, disrupts follicular steroidogenesis and oocyte maturation through lipotoxicity and oxidative stress, contributing to poor embryo quality and placental dysfunction (14).

Since these metabolic disturbances do not operate in isolation and instead interact synergistically to exacerbate PCOS-related reproductive dysfunction, integrated metabolic indicators are needed to provide a more comprehensive assessment of metabolic status. Individual metabolic parameters, such as homeostatic model assessment for IR (HOMA-IR), body mass index (BMI), and triglycerides, have been studied as potential predictors of IVF outcomes in women with PCOS (15–17). However, findings remain inconsistent and inconclusive, often failing to fully account for the interplay among metabolic factors. Recently, the triglyceride-glucose (TyG) index has gained increasing attention as a cost-effective and robust surrogate marker of IR (18). By combining TyG index with BMI, TyG-BMI not only improves its effectiveness in evaluating IR, but also reflects the obesity-related metabolic dysfunction (19).

Despite TyG-BMI’s acknowledged value in assessing metabolic risk, there have been few studies investigating the relationship between TyG-BMI and reproductive outcomes among women with PCOS undergoing IVF treatments, with most results focusing on fresh embryo transfer (ET) cycles and lack of data on obstetric and neonatal outcomes (20, 21). However, a freeze-all policy is the better choice for PCOS patients in order to reduce the risk of ovarian hyperstimulation syndrome (OHSS) (22). In addition, frozen embryo transfer (FET) provides a more physiological uterine environment than ET, avoiding the adverse effects of controlled ovarian hyperstimulation on embryo-endometrium synchronization (23, 24). To address these concerns, we conducted the present study in PCOS women who underwent their first FET cycles to investigate the effects of TyG-BMI on pregnancy, obstetric and neonatal outcomes.

## 2 Materials and methods

### 2.1 Study design and participants

This retrospective cohort study was conducted at the Center for Reproductive Medicine, Women and Children’s Hospital of Ningbo University, from January 2018 to January 2024. The study included women diagnosed with PCOS based on the Rotterdam Criteria (25), who underwent their first IVF or intracytoplasmic sperm injection (ICSI) cycle followed by FET. The diagnosis of PCOS required at least two of the following three criteria: oligomenorrhea or anovulation, clinical or biochemical hyperandrogenism, and polycystic ovaries on ultrasound; and other causes of hyperandrogenism and ovulatory dysfunction are ruled out. Exclusion criteria included: (1) women older than 40 years of age; (2) recurrent spontaneous abortion; (3) chromosome abnormality; (4) abnormal liver, renal or thyroid function; (5) uterine malformations; (6) endometriosis or adenomyosis; (7) severe male factor infertility; (8) missing important baseline or outcome data. The study was approved by the Ethics Committee of The Affiliated Women and Children’s Hospital of Ningbo University (Approval Number: EC2024-197) with a waiver for informed consent.

### 2.2 ART procedures

The controlled ovarian stimulation (COS) protocols were determined based on physician’s recommendation and patient’s preference. The gonadotropin-releasing hormone (GnRH) agonist protocol involved the administration of a short-acting GnRH agonist (Triptorelin acetate, Lummy, China; 0.1mg daily) starting at the luteal phase or a long-acting GnRH agonist (Beiyi^®^, Livzon, China; 3.75mg) on days 1–2 of the menstrual cycle. When pituitary down-regulation was achieved, ovarian stimulation was initiated by using daily injections of 100–300 IU urine-derived follicular stimulating hormone (FSH, Lishenbao^®^, Livzon, China) individualized based on age, BMI, ovarian reserve and prior response. Dose adjustments were guided by serial ultrasonography and serum hormone monitoring. Final oocyte maturation was triggered with human chorionic gonadotropin (hCG, Livzon, China; 4000–10000 IU) when ≥ 2 follicles reached mean diameter of 18 mm. The GnRH antagonist protocol consisted of daily ovarian stimulation with 100-150IU FSH from day 3–4 of the menstrual cycle. GnRH antagonist (Cetrotide^®^, Merck Serono, Switzerland; 0.25 mg daily) was initiated when leading follicles reached 11–12 mm diameter with serum estradiol (E2) approaching 1000 pg/mL. When 3 follicles reached ≥ 17 mm diameter or at least one follicle reached 18-20mm diameter, 2000 IU of hCG and 0.1mg of GnRH agonist (Decapeptyl^®^, Ferring, Germany) was administered for triggering. Retrieved oocytes were fertilized either by conventional IVF or ICSI or rescue ICSI based on semen analysis, the couple’s history and fertilization failure of IVF. Fertilization was confirmed by the presence of two pronuclei (2PN) 16–18 hours post-fertilization. Embryos were cultured in a standard medium and evaluated on day 3 (D3) as well as on day 5 (D5) and day 6 (D6), based on the number of cells, degree of fragmentation, and morphological characteristics. On Day 3 after oocyte retrieval, cleavage embryos with at least six cells and < 20% fragmentation were considered as high quality and eligible for transfer or cryopreservation. On day 5 or 6 after oocyte retrieval, blastocysts were graded by Gardner criteria (26). Blastocysts scored ≥ 3BB were considered as high-quality and scored ≥ 4BC were eligible for cryopreserved.

### 2.3 Frozen-thawed embryo transfer

Endometrial preparation was performed in either a hormone replacement cycle, a natural cycle or a stimulated cycle based on patient’s menstrual pattern and physicians’ discretion. When the endometrium reached a sufficient thickness, luteal support was added with vaginal progesterone gel (Crinone^®^, Merck Serono, Switzerland; 90 mg daily) and oral dydrogesterone (Duphaston^®^, Abbott, Netherlands; 10 mg, 3 times per day). Embryo transfer was performed under ultrasound guidance after 3 or 5 days of progesterone administration, with a maximum of two embryos transferred per cycle. Luteal support was maintained until 10 weeks of gestation if pregnancy was confirmed and can be extended if vaginal bleeding is present.

### 2.4 Exposure definitions

Blood samples were collected in the morning after overnight fasting for at least 8 hours. Fasting blood glucose (FBG), total cholesterol (TC), triglyceride (TG), low-density lipoprotein (LDL), and high-density lipoprotein (HDL) were measured using standard laboratory methods. The basal hormone blood samples were collected during the early follicular phase (cycle days 2–4). Other baseline characteristics, including age, BMI, duration of infertility, blood pressure and ovarian reserve markers (anti-müllerian hormone, AMH; antral follicle account, AFC), were also recorded before commencing IVF treatment. The TyG-BMI was calculated as follows: TyG-BMI = natural logarithm [TG (mg/dL) × FBG (mg/dL)/2] × BMI (kg/m2); BMI = weight (kg)/[height (m)]^2^ (27).

### 2.5 Outcome definitions

Biochemical pregnancy was defined as a serum β-hCG level exceeding 25 IU/L measured 14 days after embryo transfer. Clinical pregnancy was confirmed by transvaginal ultrasound visualization of at least one intrauterine gestational sac at 4–5 weeks of gestation. Miscarriage is defined as a pregnancy loss before 24 weeks of gestation. Live birth was recorded as the delivery of a viable infant at ≥ 24 weeks of gestation. Obstetric complications included GDM (10th revision of the International Statistical Classification of Diseases and Related Health Problems [ICD-10] code O24.4), hypertensive disorders of pregnancy (HDP, including pregnancy induced hypertension [ICD-10 code O13] and pre-eclampsia [ICD-10 codes O14-15]) and placental disorders (including placenta previa [ICD-10 code O44], placental abruption [ICD-10 code O45] and placenta accreta [ICD-10 code O43.2]). Neonatal outcomes encompassed gestational age, preterm birth (PTB), birth weight, LBW, macrosomia, small for gestational age (SGA), LGA and birth defects (ICD-10 codes Q00-Q99). PTB was defined as live birth before 37 gestational weeks. Low birth weight was defined as birth weight < 2500g. Macrosomia was defined as birth weight ≥ 4000g. SGA and LGA were respectively defined as birth weight < 10th percentile and > 90th percentile of gender-specific birthweight reference at the same gestational age (28).

### 2.6 Statistical analysis

Continuous data were expressed as median and interquartile range (25th-75th), whereas categorical data were expressed as frequencies (n) and percentages (%). Differences between groups were analyzed using Kruskal Wallis H test for continuous variables, and chi-square test or Fisher’s exact test for categorical variables. A trend test across TyG-BMI quartiles was performed by modeling quartiles as an ordinal variable (Q1–Q4) in regression analyses. Logistic regression models were employed to investigate the potential association between TyG-BMI and various outcomes. The adjustment models were as follows: (1) no variable was adjusted for in model 1; (2) age and duration of infertility were adjusted for in model 2; and (3) age, duration of infertility, AMH, systolic blood pressure (SBP), COS protocols, total gonadotropin (Gn) dose, duration of Gn, TC, the number of transferred embryos, the developmental stage of transferred embryos and endometrium thickness were adjusted for in model 3. Odds ratios (ORs) were calculated with 95% confidence intervals (CIs). Restricted cubic spline (RCS) models were employed to explore non-linear relationships and dose-response relationships between TyG-BMI and various outcomes. Stratified analysis was performed to assess the impact of female age (≤ 30 and > 30 years), BMI (< 24, 24~28, and ≥ 28 kg/m^2^) and AMH levels (low and high) on these relationships. Furthermore, the least absolute shrinkage and selection operator (LASSO) regression model was used to identify potential risk variables for miscarriage and receiver operating characteristic (ROC) curve analysis was conducted to evaluate the predictive power. All statistical analyses were performed using R software (version 4.4.1). All P values were two-sided, and P < 0.05 was considered statistically significant.

## 3 Results

### 3.1 Demographic and cycle characteristics

A total of 744 PCOS patients were included for analysis. The participants were categorized into four groups according to TyG-BMI quartiles (Quartile 1 (Q1): < 172.6, Quartile 2 (Q2): 172.6-197.5, Quartile 3 (Q3): 197.5-225.0, Quartile 4 (Q4): ≥ 225.0). The baseline characteristics of these groups are presented in **Table 1**. Compared with women in the lower TyG-BMI quartiles, women in the higher quartiles tend to be older, have higher BMI, SBP, diastolic blood pressure (DBP), TG, TC, LDL and FBG, longer infertility duration, but lower AMH, basal FSH, basal luteinizing hormone (LH) and HDL (all P trend < 0.05). The level of basal E2 was also shown a significant difference across the groups (P < 0.05).

**Table 1:** Baseline characteristics of the participants according to TyG-BMI quartiles in polycystic ovary syndrome.

Variables	Overall N = 744	Quantile 1 N = 186	Quantile 2 N = 186	Quantile 3 N = 186	Quantile 4 N = 186	P trend	P value
Age (year)	29.0 (27.0, 32.0)	29.0 (27.0, 31.0)	29.0 (27.0, 31.0)	30.0 (27.0, 32.0)	30.0 (27.0, 32.0)	0.014	0.060
BMI (kg/m2)	22.9 (20.6, 25.4)	19.3 (18.6, 20.3)	21.8 (20.8, 22.6)	24.1 (23.1, 25.0)	27.3 (26.2, 29.1)	<0.001	<0.001
SBP (mm Hg)	117.0 (110.0, 125.0)	113.0 (106.0,121.0)	116.0 (108.0,125.0)	119.0 (112.0,125.0)	121.0 (113.0,129.0)	<0.001	<0.001
DBP (mm Hg)	72.0 (66.0, 78.0)	69.0 (63.0,74.0)	72.0 (65.0,77.0)	72.0 (66.0,78.0)	76.0 (70.0,81.0)	<0.001	<0.001
Duration of infertility (year)	3.0 (2.0, 5.0)	3.0 (2.0, 4.0)	3.0 (2.0, 4.0)	3.0 (2.0, 4.0)	4.0 (2.0, 5.0)	<0.001	<0.001
Primary infertility, n (%)	527 (70.8%)	138 (74.2%)	121 (65.1%)	128 (68.8%)	140 (75.3%)	0.639	0.104
Basal AFC, n	20.0 (16.0, 20.0)	20.0 (16.0, 20.0)	20.0 (16.0, 20.0)	20.0 (16.0, 20.0)	20.0 (18.0, 20.0)	0.128	0.073
AMH (ng/ml)	9.0 (6.0, 13.3)	10.6 (7.1, 14.9)	10.3 (6.9, 14.1)	8.5 (5.8, 12.5)	7.6 (4.8, 11.4)	<0.001	<0.001
Basal FSH (IU/L)	6.1 (5.0, 7.5)	6.6 (5.3, 7.6)	6.1 (5.0, 7.7)	6.0 (4.9, 7.6)	5.6 (4.9, 6.7)	0.004	0.010
Basal LH (IU/L)	5.2 (2.3, 8.9)	6.2 (2.6, 12.5)	5.3 (2.1, 7.9)	5.5 (2.4, 8.5)	4.3 (2.0, 8.1)	<0.001	0.006
Basal E2 (pg/ml)	37.2 (26.0, 61.0)	40.5 (28.0, 62.0)	41.0 (26.0, 83.5)	35.8 (24.0, 57.0)	34.3 (26.0, 56.0)	0.152	0.035
Basal T (ng/dl)	50.4 (36.4, 67.0)	50.4 (36.3, 68.5)	48.8 (37.0, 65.7)	48.4 (34.3, 64.5)	52.6 (38.8, 69.0)	0.560	0.515
Basal PRL (ng/ml)	12.4 (9.0, 17.1)	12.9 (8.7, 18.4)	12.5 (9.5, 17.4)	12.6 (9.6, 16.0)	11.2 (8.3, 16.5)	0.203	0.139
TG (mmol/L)	1.4 (1.0, 2.0)	0.9 (0.7, 1.2)	1.2 (1.0, 1.6)	1.6 (1.3, 2.4)	2.0 (1.5, 2.7)	<0.001	<0.001
TC (mmol/L)	4.8 (4.2, 5.3)	4.5 (4.1, 5.2)	4.7 (4.1, 5.2)	4.9 (4.2, 5.4)	4.9 (4.5, 5.4)	<0.001	0.002
HDL (mmol/L)	1.5 (1.2, 1.8)	1.7 (1.5, 2.0)	1.6 (1.3, 1.8)	1.4 (1.2, 1.6)	1.2 (1.1, 1.4)	<0.001	<0.001
LDL (mmol/L)	2.5 (2.0, 2.9)	2.3 (1.9, 2.6)	2.4 (2.0, 2.8)	2.5 (2.1, 3.0)	2.7 (2.2, 3.0)	<0.001	<0.001
FBG (mmol/L)	5.0 (4.7, 5.4)	5.0 (4.6, 5.2)	4.9 (4.7, 5.3)	5.1 (4.8, 5.4)	5.2 (4.9, 5.5)	<0.001	<0.001

Data are presented as median (interquartile range) or number (percentage).

TyG-BMI, triglyceride glucose-body mass index; BMI, body mass index; SBP, systolic blood pressure; DBP, diastolic blood pressure; AFC, antral follicle account; AMH, anti-müllerian hormone; FSH, follicle-stimulating hormone; LH, luteinizing hormone; E2, estradiol; T, testosterone; PRL, prolactin; TG, triglycerides; TC, total cholesterol; HDL, high-density lipoprotein; LDL, low-density lipoprotein; FBG, fasting blood glucose.

The COS protocols and laboratory outcomes were shown in **Table 2**. A significantly higher proportion of GnRH agonist cycles was observed in the Q2-Q4 groups compared to the Q1 group (P trend < 0.05). The starting Gn dose, total Gn dose and duration of Gn increased significantly across TyG-BMI quartiles (all P trend < 0.05). Notably, patients in higher quantiles tended to retrieve fewer oocytes, with the median count decreasing from 16.0 in Q1 to 14.0 in Q4 (P trend = 0.008). Likewise, there was a significant decline in the quantity of metaphase II (MII) oocytes, 2PN zygotes and cleavage embryos from Q1 to Q4 group (all P trend < 0.05). However, no differences were observed in the quantity of high-quality embryos or blastocyst formation rates (all P > 0.05). Fertilization methods, endometrial thickness on the day of embryo transfer, embryo developmental stage at transfer and the number of embryos transferred did not differ significantly among the groups (all P > 0.05).

**Table 2:** Ovarian stimulation, *in vitro* fertilization, and related outcomes according to TyG-BMI quartiles in polycystic ovary syndrome.

Variables	Overall N=744	Quantile 1 N = 186	Quantile 2 N = 186	Quantile 3 N = 186	Quantile 4 N = 186	P trend	P value
COS protocol, n (%)						0.008	0.003
GnRH agonist	601 (80.8%)	133 (71.5%)	155 (83.3%)	161 (86.6%)	152 (81.7%)		
GnRH antagonist	137 (18.4%)	51 (27.4%)	28 (15.1%)	25 (13.4%)	33 (17.7%)		
Other	6 (0.8%)	2 (1.1%)	3 (1.6%)	0 (0.0%)	1 (0.5%)		
Starting dose of Gn (IU)	225.0 (187.5, 225.0)	187.5 (150.0, 187.5)	187.5 (187.5, 225.0)	225.0 (187.5, 225.0)	225.0 (225.0, 300.0)	<0.001	<0.001
Total Gn dose (IU)	2250.0 (1837.5, 2962.5)	1837.5 (1500.0, 2250.0)	2025.0 (1725.0, 2550.0)	2418.8 (2025.0, 3037.5)	3037.5 (2362.5, 3787.5)	<0.001	<0.001
Duration of Gn (day)	11.0 (9.0, 12.0)	10.0 (9.0, 11.0)	10.0 (9.0, 11.0)	11.0 (10.0, 12.0)	11.0 (10.0, 13.0)	<0.001	<0.001
No. of oocytes retrieved (n)	15.0 (11.0, 20.0)	16.0 (12.0, 21.0)	15.0 (11.0, 20.0)	15.0 (11.0, 19.0)	14.0 (10.0, 19.0)	0.008	0.048
No. of MII oocytes (n)	13.0 (10.0, 18.0)	14.0 (10.0, 19.0)	13.0 (9.0, 18.0)	13.0 (10.0, 17.0)	12.0 (9.0, 17.0)	0.004	0.008
No. of 2PN zygotes (n)	10.0 (6.0, 14.0)	11.0 (7.0, 14.0)	10.0 (7.0, 13.0)	10.0 (7.0, 14.0)	9.0 (6.0, 13.0)	0.026	0.035
No. of cleavage embryos (n)	11.0 (8.0, 16.0)	12.0 (9.0, 17.0)	11.0 (8.0, 15.0)	12.0 (8.0, 16.0)	11.0 (6.0, 15.0)	0.021	0.034
No. of high-quality embryos (n)	7.0 (4.0, 10.0)	7.5 (4.0, 11.0)	7.0 (4.0, 9.0)	7.5 (4.0, 10.0)	6.0 (4.0, 10.0)	0.136	0.111
Blastocyst formation rate (%)	60.0 (40.0, 75.0)	61.7 (45.5, 76.9)	60.0 (38.5, 75.0)	57.1 (40.0, 71.4)	57.1 (40.0, 75.0)	0.236	0.439
Fertilization, n (%)						0.558	0.825
IVF	670 (90.1%)	170 (91.4%)	168 (90.3%)	166 (89.2%)	166 (89.2%)		
ICSI	51 (6.9%)	9 (4.8%)	14 (7.5%)	15 (8.1%)	13 (7.0%)		
IVF/ICSI	23 (3.1%)	7 (3.8%)	4 (2.2%)	5 (2.7%)	7 (3.8%)		
Endometrial thickness on the day of embryo transfer (mm)	9.0 (8.0, 10.0)	9.0 (8.0, 10.0)	9.0 (8.0, 10.0)	9.0 (8.0, 10.0)	9.0 (8.0, 10.0)	0.149	0.468
Embryo developmental stage at transfer, n (%)						0.074	0.312
Cleavage	152 (20.4%)	31 (16.7%)	35 (18.8%)	43 (23.1%)	43 (23.1%)		
Blastocyst	592 (79.6%)	155 (83.3%)	151 (81.2%)	143 (76.9%)	143 (76.9%)		
Transferred embryos, n (%)						0.238	0.496
Single	461 (62.0%)	124 (66.7%)	113 (60.8%)	111 (59.7%)	113 (60.8%)		
Double	283 (38.0%)	62 (33.3%)	73 (39.2%)	75 (40.3%)	73 (39.2%)		

Data are presented as median (interquartile range) or number (percentage).

TyG-BMI, triglyceride glucose-body mass index; COS, controlled ovarian stimulation; GnRH, gonadotropin-releasing hormone; Gn, gonadotropin; MII, metaphase II; 2PN, two pronucleus; IVF, *in vitro* fertilization; ICSI, intracytoplasmic sperm injection.

### 3.2 Pregnancy outcomes

A total of 410 (55.1%) women achieved live births following their FET cycles. The pregnancy outcomes and their associations with TyG-BMI levels assessed by univariate and multivariate analyses were summarized in **Table 3**. Notably, there was a significant increasing trend in miscarriage rate from Q1 (8.9%) to Q4 (27.9%), and the logistic regression analyses showed stable positive correlations between TyG-BMI and miscarriage rate in all three models (all P trend < 0.001). Compared with Q1, women in Q3 (OR, 1.13; 95% CI, 1.02–1.25) and Q4 (OR, 1.26; 95% CI, 1.12–1.41) showed a significantly higher risk of miscarriage after full adjustment for covariates. The live birth rate decreased significantly from Q1 (60.8%) to Q4 (47.3%) and was negatively correlated with TyG-BMI in all three models (all P trend < 0.05). Compared with Q1, women in Q4 had a significantly lower probability of live birth after fully adjusted (OR, 0.86; 95% CI, 0.76-0.97). However, no statistically significant associations were observed between TyG-BMI and the rates of biochemical pregnancy and clinical pregnancy following the first FET in all three regression models (all P trend > 0.05).

**Table 3:** Pregnancy outcomes and their associations with TyG-BMI in polycystic ovary syndrome.

Outcomes	Overall N=744	Quantile 1 N = 186	Quantile 2 N = 186	Quantile 3 N = 186	Quantile 4 N = 186	P trend
Biochemical pregnancy						
n (%)	558 (75.0%)	140 (75.3%)	144 (77.4%)	137 (73.7%)	137 (73.7%)	
Model 1 OR (95%CI)	–	Ref.	1.02 (0.94-1.12)	0.98 (0.90-1.07)	0.98 (0.90-1.07)	0.546
Model 2 OR (95%CI)	–	Ref.	1.03 (0.94-1.12)	1.00 (0.91-1.09)	1.00 (0.91-1.09)	0.826
Model 3 OR (95%CI)	–	Ref.	1.02 (0.93-1.11)	0.97 (0.88-1.06)	0.93 (0.84-1.04)	0.132
Clinical pregnancy						
n (%)	499.0 (67.1%)	124 (66.7%)	125 (67.2%)	128 (68.8%)	122 (65.6%)	
Model 1 OR (95%CI)	–	Ref.	1.01 (0.91-1.11)	1.02 (0.93-1.12)	0.99 (0.90-1.09)	0.917
Model 2 OR (95%CI)	–	Ref.	1.01 (0.92-1.11)	1.03 (0.94-1.14)	1.01 (0.92-1.11)	0.729
Model 3 OR (95%CI)	–	Ref.	1.01 (0.91-1.11)	1.03 (0.93-1.14)	0.99 (0.88-1.12)	0.973
Miscarriage						
n (%)	89.0 (17.8%)	11 (8.9%)	21 (16.8%)	23 (18.0%)	34 (27.9%)	
Model 1 OR (95%CI)	–	Ref.	1.08 (0.99-1.19)	1.10 (1.00-1.20)	1.21 (1.10-1.33)	<0.001
Model 2 OR (95%CI)	–	Ref.	1.08 (0.98-1.19)	1.09 (1.00-1.20)	1.21 (1.10-1.33)	<0.001
Model 3 OR (95%CI)	–	Ref.	1.10 (1.00-1.22)	1.13 (1.02-1.25)	1.26 (1.12-1.41)	<0.001
Live birth						
n (%)	410.0 (55.1%)	113 (60.8%)	104 (55.9%)	105 (56.5%)	88 (47.3%)	
Model 1 OR (95%CI)	–	Ref.	0.95 (0.86-1.05)	0.96 (0.87-1.06)	0.87 (0.79-0.97)	0.015
Model 2 OR (95%CI)	–	Ref.	0.95 (0.86-1.06)	0.97 (0.88-1.07)	0.89 (0.80-0.98)	0.041
Model 3 OR (95%CI)	–	Ref.	0.95 (0.85-1.05)	0.94 (0.84-1.05)	0.86 (0.76-0.97)	0.020

TyG-BMI, triglyceride glucose-body mass index; OR, odds ratio; CI, confidence interval.

Model 1, unadjusted model; Model 2, adjusted for age and duration of infertility; Model 3, adjusted for age, duration of infertility, anti-müllerian hormone, systolic blood pressure, controlled ovarian stimulation protocols, total gonadotropin (Gn) dose, duration of Gn, total cholesterol, the number of transferred embryos, the developmental stage of transferred embryos and endometrium thickness.

### 3.3 Obstetric and neonatal outcomes

There were 360 singletons (78.3%) and 100 twins (21.7%) born during the study period. The obstetric complications and neonatal outcomes stratified by TyG-BMI quartiles are presented in **Table 4**. The incidence of GDM increased from 9.7% in Q1 to 35.2% in Q4 (P trend < 0.001), with parallel increases observed in macrosomia rates (Q1: 4.8% vs Q4: 12.2%, P trend = 0.036) and LGA rates (Q1: 12.7% vs Q4: 32.7%, P trend < 0.001). Singleton birth weights in Q4 exceeded those in Q1–Q3 (P = 0.048), whereas twin birth weights remained comparable across quartiles (P > 0.05). Low birth weight incidence demonstrated a nonsignificant upward trend from Q1 (12.7%) to Q4 (18.4%) (P trend = 0.257). No statistical differences were noticed amongst TyG-BMI quartiles in HDP, placental disorders, gestational age, PTB, SGA and birth defect (all P trend > 0.05).

**Table 4:** Perinatal outcomes according to TyG-BMI quartiles in polycystic ovary syndrome.

Variables	Overall N=744	Quantile 1 N = 186	Quantile 2 N = 186	Quantile 3 N = 186	Quantile 4 N = 186	P trend	P value
No. of live birth	460	126	121	115	98		
Singleton	360 (78.3%)	100 (79.4%)	87 (71.9%)	95 (82.6%)	78 (79.6%)		
Twins	100 (21.7%)	26 (20.6%)	34 (28.1%)	20 (17.4%)	20 (20.4%)		
GDM, n (%)	98 (23.7%)	11 (9.7%)	21 (20.2%)	31 (29.5%)	31 (35.2%)	<0.001	<0.001
HDP, n (%)	28 (6.8%)	4 (3.5%)	7 (6.7%)	8 (7.6%)	9 (10.2%)	0.065	0.305
Placental Disorders, n (%)	20 (4.9%)	5 (4.4%)	4 (3.8%)	7 (6.7%)	4 (4.5%)	0.743	0.827
Gestational age (week)	38.0 (37.0, 39.0)	39.0 (37.0, 39.0)	38.0 (37.0, 39.0)	38.0 (37.0, 39.0)	38.0 (37.0, 39.0)	0.072	0.234
Preterm birth, n (%)	71 (17.3%)	15 (13.3%)	20 (19.2%)	19 (18.1%)	17 (19.3%)	0.318	0.605
Birth weight (g)	3250.0 (2800.0, 3600.0)	3225.0 (2800.0, 3650.0)	3200.0 (2800.0, 3450.0)	3240.0 (2840.0, 3600.0)	3400.0 (2800.0, 3700.0)	0.154	0.078
Singleton	3400.0 (3100.0, 3700.0)	3350.0 (3060.0, 3735.0)	3350.0 (3150.0, 3600.0)	3350.0 (3000.0, 3650.0)	3550.0 (3250.0, 3830.0)	0.141	0.048
Twins	2525.0 (2025.0, 2800.0)	2525.0 (2025.0, 2700.0)	2550.0 (1900.0, 2775.0)	2712.5 (1165.0, 2815.0)	2430.0 (2200.0, 2750.0)	0.949	0.992
LBW, n (%)	65 (14.1%)	16 (12.7%)	16 (13.2%)	15 (13.0%)	18 (18.4%)	0.257	0.603
Macrosomia, n (%)	38 (8.3%)	6 (4.8%)	9 (7.4%)	11 (9.6%)	12 (12.2%)	0.036	0.216
SGA, n (%)	11 (2.4%)	5 (4.0%)	3 (2.5%)	1 (0.9%)	2 (2.0%)	0.256	0.491
LGA, n (%)	91 (19.8%)	16 (12.7%)	20 (16.5%)	23 (20.0%)	32 (32.7%)	<0.001	0.002
Birth defect, n (%)	8 (1.7%)	1 (0.8%)	2 (1.7%)	2 (1.7%)	3 (3.1%)	0.216	0.633

TyG-BMI, triglyceride glucose-body mass index; GDM, gestational diabetes mellitus; HDP, hypertensive disorders of pregnancy; LBW, low birthweight; SGA, small for gestational age; LGA, large for gestational age.

**Table 5** presents the associations between TyG-BMI quartiles and perinatal outcomes using regression analyses with or without adjustments. The incidence of GDM was positively associated with TyG-BMI quartiles in all three models (all P trend < 0.05). In the fully adjusted model, women in Q3 (OR 1.18, 95%CI 1.04-1.33) and Q4 (OR 1.18, 95%CI 1.03-1.36) showed a 18% elevated risk of GDM compared with those in Q1. Notably, the risk of LGA showed a positive correlation with TyG-BMI after adjusting for maternal age and duration of infertility (P trend < 0.001), with a 21% increased risk of LGA in Q4 compared with that in Q1 (OR 1.21, 95%CI 1.09-1.35). However, with further adjustment for SBP, AMH, TC, COS protocols, total Gn dose, duration of Gn, the number of embryos transferred, the developmental stage of transferred embryos and endometrial thickness in model 3, no significant correlation was found between the incidence of LGA and TyG-BMI (P trend > 0.05). In addition, the incidences of PTB and LBW remained consistently nonsignificant across all models (all P trend > 0.05).

**Table 5:** Associations between TyG-BMI and perinatal outcomes in polycystic ovary syndrome.

Outcomes	OR (95%CI)				P trend
	Quantile 1	Quantile 2	Quantile 3	Quantile 4	
GDM					
Model 1	Ref	1.12 (1.00-1.25)	1.23 (1.11-1.38)	1.30 (1.16-1.46)	<0.001
Model 2	Ref	1.12 (1.00-1.25)	1.23 (1.10-1.37)	1.29 (1.15-1.45),	<0.001
Model 3	Ref	1.10 (0.98-1.24)	1.18 (1.04-1.33)	1.18 (1.03-1.36)	0.014
PTB					
Model 1	Ref	1.06 (0.96-1.17)	1.05 (0.95-1.16)	1.06 (0.96-1.18)	0.318
Model 2	Ref	1.06 (0.96-1.17)	1.05 (0.95-1.16)	1.07 (0.96-1.19)	0.300
Model 3	Ref	1.02 (0.92-1.13)	1.00 (0.89-1.11)	1.00 (0.88-1.14)	0.935
LBW					
Model 1	Ref	1.01 (0.92-1.10)	1.00 (0.92-1.10)	1.06 (0.97-1.16)	0.257
Model 2	Ref	1.01 (0.92-1.10)	1.00 (0.92-1.10)	1.06 (0.96-1.16)	0.282
Model 3	Ref	0.97 (0.89-1.05)	0.98 (0.90-1.08)	1.06 (0.95-1.18)	0.253
LGA					
Model 1	Ref	1.04 (0.94-1.15)	1.08 (0.97-1.19)	1.22 (1.10-1.35)	<0.001
Model 2	Ref	1.04 (0.94-1.15)	1.08 (0.98-1.19)	1.21 (1.09-1.35)	<0.001
Model 3	Ref	1.02 (0.92-1.13)	1.04 (0.94-1.16)	1.13 (0.99-1.28)	0.063

TyG-BMI, triglyceride glucose-body mass index; OR, odds ratio; CI, confidence interval; GDM, gestational diabetes mellitus; PTB, preterm birth; LBW, low birth weight; LGA, large for gestational age; Ref, reference.

Model 1, unadjusted model; Model 2, adjusted for age and duration of infertility; Model 3, adjusted for age, duration of infertility, anti-müllerian hormone, systolic blood pressure, controlled ovarian stimulation protocols, total gonadotropin (Gn) dose, duration of Gn, total cholesterol, the number of transferred embryos, the developmental stage of transferred embryos and endometrium thickness.

### 3.4 The analyses of non-linear relationship

**Figure 1:**
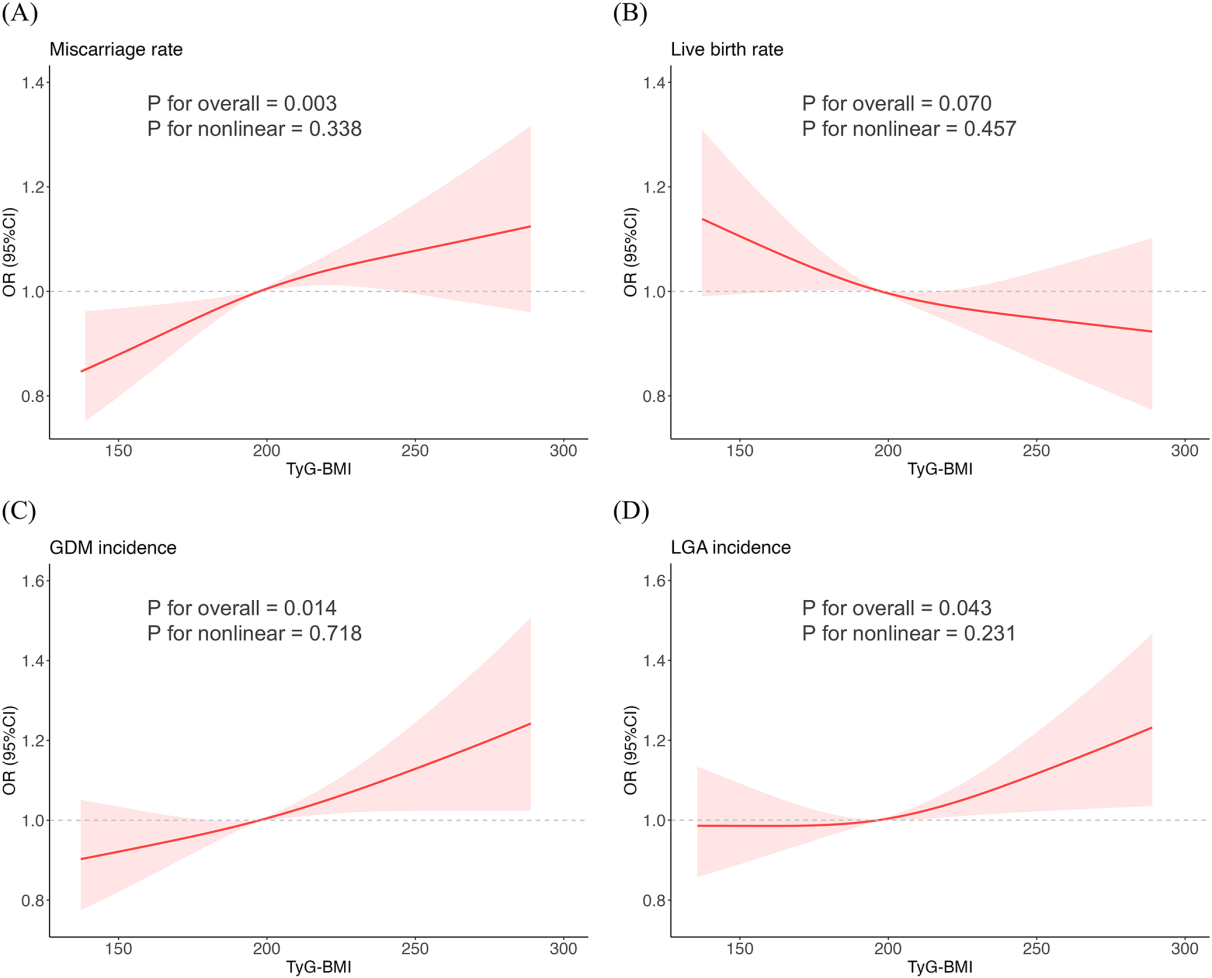
Restricted cubic spline fitting for the association between TyG-BMI and reproductive outcomes. **(A)** Miscarriage rate. **(B)** Live birth rate. **(C)** GDM incidence. **(D)** LGA incidence.

Considering the continuous nature of TyG-BMI, we employed RCS models to analyze its non-linear relationships with various outcomes in our study (**Figure 1**). After full adjustment for covariates in the master analytical model 3 above, significant linear correlations were observed between TyG-BMI and miscarriage rate, GDM incidence and LGA incidence (P for nonlinear > 0.05, P for overall < 0.05). However, no significant association was found between TyG-BMI and live birth rate (P for nonlinear > 0.05, P for overall > 0.05).

### 3.5 Stratified analyses

Stratified analyses were conducted to evaluate the impact of potential confounding factors on the relationship between TyG-BMI and reproductive outcomes. As shown in **Supplementary Figure S1**, no interaction was found between TyG-BMI and stratification factors on the associations with miscarriage rate, live birth rate, the risk of GDM and the risk of LGA (all P for interaction >0.05).

### 3.6 ROC analyses for predicting miscarriage

**Figure 2:**
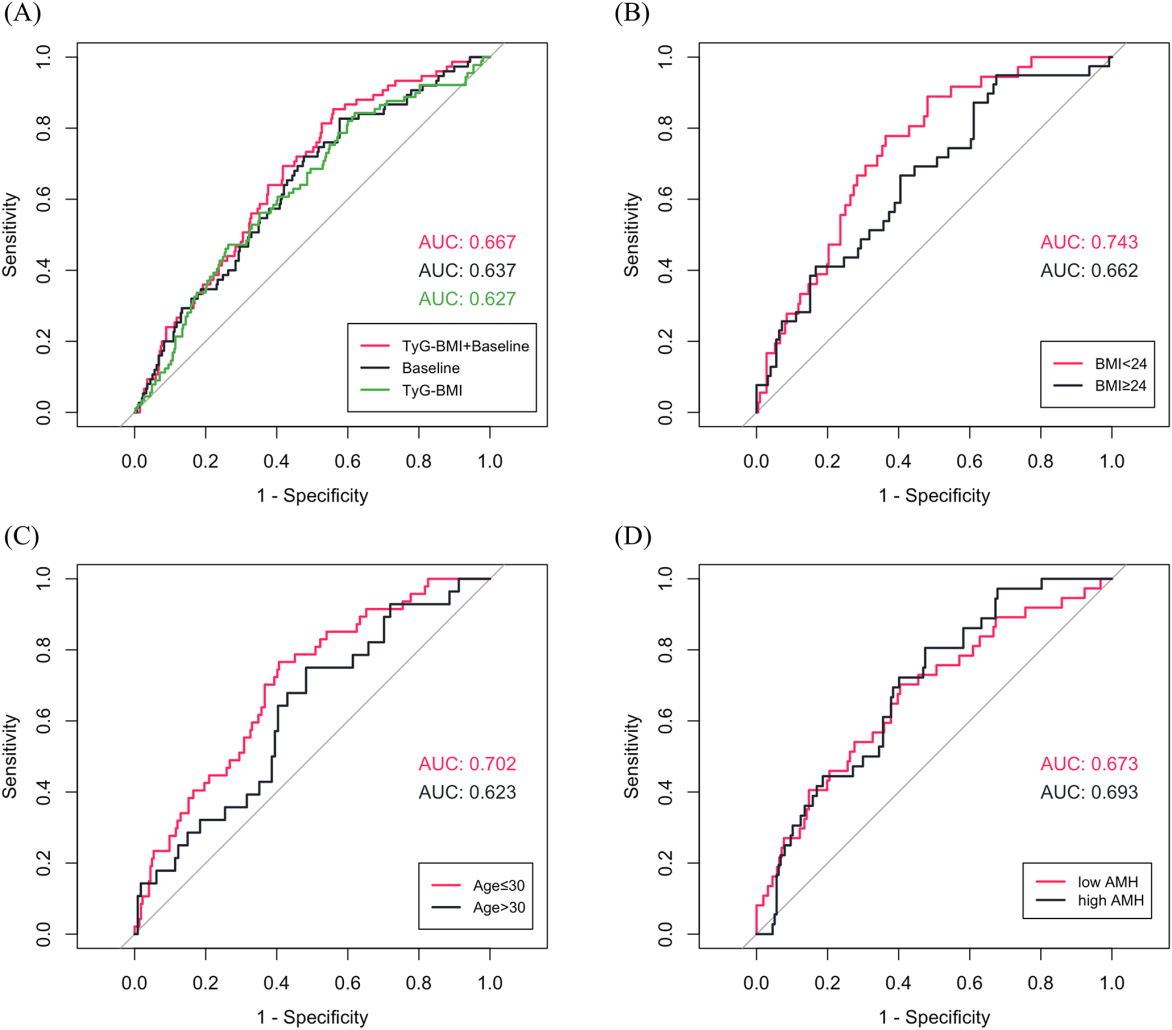
**(A)** ROC curves for predicting miscarriage in total patients (Baseline characteristics: LH, FSH, total cholesterol, testosterone, infertility type and controlled ovarian stimulation protocols). **(B-D)** ROC curves of TyG-BMI combined with baseline characteristics for predicting miscarriage in subgroups.

Using LASSO regression, seven variables were identified as independent predictors of miscarriage: TyG-BMI, basal LH, basal FSH, TC, T, infertility type (primary vs. secondary) and COS protocols. The coefficient track diagram and the ten-fold cross-validation error curve were provided in **Supplementary Figure S2**. ROC curve analyses demonstrated that TyG-BMI alone was predictive of miscarriage (area under the curve (AUC) = 0.627, P < 0.001) with a cutoff value of 180.4. The full model integrating TyG-BMI with baseline characteristics (LH, FSH, TC, T, infertility type and COS protocols) achieved higher discriminative power than other two models (AUC = 0.667, P < 0.001) (**Figure 2A**, **Supplementary Table S1**). And the full model showed good miscarriage prediction performance in most subgroups (AUC > 0.650), particularly in patients with normal/low weight (BMI < 24 kg/m^2^, AUC = 0.743, P < 0.001) or young age (Age ≤ 30, AUC = 0.702, P < 0.001) (**Figures 2B–D**, **Supplementary Table S2**).

## 4 Discussion

This study is the first to systematically explore the impact of TyG-BMI on pregnancy outcomes, obstetric complications and neonatal outcomes following the first FET in patients with PCOS. The results showed that an elevated TyG-BMI was independently associated with an increased risk of miscarriage, a decreased probability of live birth, and an increased risk of GDM and LGA. These findings suggest that TyG-BMI is not only a predictor of pregnancy outcomes in patients with PCOS, but also a potential early warning marker for long-term maternal and infant health.

IR is prevalent in women with PCOS, which is strongly associated with metabolic and reproductive complications (11). In contrast to the time-consuming and complex hyperinsulin-normoglycemic clamp technique, which is considered the gold standard for diagnosing IR, HOMA-IR serves as an alternative biomarker of IR (29). However, fasting insulin levels are not routinely measured in clinical practice, which may miss the optimal time to intervene in various metabolic risk factors. In contrast, the TyG-BMI, with its multiple advantages of low cost, easy accessibility, independence from insulin therapy, and more comprehensive assessment of metabolic health, not only correlates strongly with HOMA-IR, but also better predicts metabolic diseases such as diabetes and cardiovascular disease (27, 30, 31). It has been shown that the TyG-BMI is a clinically convenient and practical marker for the early identification of IR and metabolic syndrome in patients with PCOS (32, 33). However, its correlation with the IVF outcomes in women with PCOS has not been fully investigated. Li et al. (20) reported that higher TyG-BMI was associated with fewer available embryos and reduced high-quality embryos, but not significantly correlated with the pregnancy outcomes of fresh ET. However, due to the higher risk of OHSS in patients with PCOS, the strategy of whole embryo freezing and subsequent FET would be more commonly used in clinical practice, resulting in a limited number of fresh ET cycles in this study (only 49 fresh ET cycles out of 966 IVF cycles), which limits the reliability of the results. Subsequently, a study by Wu et al. (21) comparing the correlation between different surrogate indicators of IR and IVF outcomes in patients with PCOS, which included a total of 358 fresh ET cycles and 185 FET cycles, showed that TyG-BMI, TyG, and HOMA-IR were negatively correlated with the live birth rate in the fresh ET cycles, and that the predictive efficacy of TyG-BMI was superior than TyG and HOMA-IR in terms of the live birth rate. However, this relationship did not exist in FET cycles. In contrast, our study, which included PCOS patients with freeze-all strategy, further controlled for confounders and expanded the sample size (a total of 744 FET cycles), found that an elevated TyG-BMI was independently associated with an increased miscarriage rate and a decreased live birth rate after the first FET in PCOS patients. Compared with the lowest TyG-BMI quartile (TyG-BMI < 172.6), the highest quartile (TyG-BMI ≥ 225.0) showed an 19% higher rate of miscarriage and a 13.5% lower rate of live birth. This trend persisted after adjustment for confounding factors.

Miscarriage is the most common adverse pregnancy outcome, affecting 15% of recognized pregnancies (34). It not only leads to both physical (e.g., hemorrhage, infection) and psychological (e.g., anxiety, depression and elevated suicide risk) detriments, but may also serve as a sentinel risk marker for obstetric complications (e.g., preterm birth, placental abruption) (35–37). PCOS has long been suggested to have an independent association with miscarriage, with some research reporting rates as high as 20%–45% for spontaneous miscarriage (38). Therefore, it is critical to identify potential risk factors and develop a low-cost and efficient prediction model based on routine clinical parameters to optimize pregnancy management for patients with PCOS. In our study, we identified for the first time that TyG-BMI was a potential predictor of miscarriage in FET among patients with PCOS, with a cutoff value of 180.4 (AUC = 0.627, P < 0.001). In addition, the potential risk factors included the serum levels of basal LH, basal FSH, TC and T, infertility type and COS protocols, which were consistent with previous related studies (39–44). The baseline model incorporating risk factors other than TyG-BMI achieved an AUC of 0.637. After adding TyG-BMI to the model, the AUC increased to 0.667 and this full model had a good predictive performance in most subgroups (AUC > 0.650, P < 0.05). Notably, the full model had the highest AUC value for predicting miscarriage in the normal or low weight population (BMI < 24 kg/m^2^, AUC = 0.743, P < 0.001), where metabolic dysregulation is often overlooked. It also indicates heterogeneous etiologies of miscarriage across BMI strata (AUC = 0.662 in the population with a BMI ≥ 24 kg/m^2^). For obese PCOS patients, miscarriage risk may be more multifactorial, involving mechanisms that are not fully captured by the current model variables, such as chronic low-grade inflammation (45). Future studies should explore additional biomarkers to refine the predictive model.

Maternal metabolic disorders not only impair oocyte quality by affecting the ovarian microenvironment, but may also affect endometrial and placental function, thereby increasing the risk of gestational complications and adverse birth outcomes (46–48). Emerging evidence indicates that early-pregnancy TyG-BMI is associated with the risk of gestational complications and adverse birth outcomes. Meng et al. (49) studied 1136 singleton pregnancies and found that the first-trimester TyG-BMI was strongly associated with the risk of HDP, GDM, and LGA. The study also analyzed TyG-BMI trajectories throughout pregnancy and found that all three observed TyG-BMI trajectories increased over time, suggesting the need for early assessment of TyG-BMI. However, the impact of pre-pregnancy TyG-BMI on maternal and neonatal health has not been studied. Moreover, ART-related techniques (including ovarian stimulation, *in vitro* embryo culture, cryopreservation, endometrial preparation protocols, and embryo transfer) and PCOS are risk factors for adverse perinatal outcomes, highlighting the need for early screening and intervention in women with PCOS undergoing IVF to improve maternal and neonatal health (50–53). Therefore, our study represents the first analysis for the association of pre-pregnancy TyG-BMI with obstetric and neonatal outcomes after FET in patients with PCOS, and the results showed that the highest TyG-BMI quartile group had a 25.5% higher incidence of GDM, a 6.7% higher incidence of HDP, and a 20% higher incidence of LGA compared to the lowest quartile group. After adjusting for confounders, TyG-BMI was positively associated with the risk of GDM and LGA, but not significantly associated with HDP, which may be related to its low incidence in our study.

To the best of our knowledge, the present study has several strengths. First, based on a large sample size, this is the first study that revealed the independent predictive value of TyG-BMI in IVF-FET outcomes in women with PCOS, suggesting that PCOS patients with elevated TyG-BMI may have a higher risk of miscarriage and a lower probability of live birth, and that metabolic optimization (e.g., metformin treatment, lifestyle interventions) should be recommended prior to IVF to improve the pregnancy outcomes. Second, we included obstetric and neonatal outcomes in the analysis and revealed for the first time the correlation between pre-conception TyG-BMI and the risk of GDM and LGA after FET in PCOS patients, suggesting that metabolic health tracking during pregnancy and maternal and infant health follow-up should be strengthened in PCOS patients with higher TyG-BMI before IVF, which provided a more comprehensive clinical perspective for the study. In addition, we applied multivariate logistic regression models to fully adjust for confounders, profiled the nonlinear relationships, and performed stratified interaction analysis to enhance the reliability of the results. Inevitably, this study has some limitations. First, due to the single-center retrospective design, selection bias should not be ignored, although we have reduced the effects of confounding factors by strict inclusion and exclusion criteria. Second, residual confounding from unmeasured factors (e.g., diet, physical activity) may have affected the results to some extent. In addition, although the FET strategy excludes the potential effects of ovarian stimulation on the endometrium, the TyG-BMI was measured before COS and fluctuations before FET were not tracked. It might have led to overlook of potential variations in TyG-BMI values before FET due to metabolic interventions such as medication and weight loss. Therefore, our findings should be interpreted with caution, and prospective cohort studies—particularly those evaluating metabolic interventions guided by TyG-BMI—are needed to confirm our results and optimize clinical outcomes in the future.

## 5 Conclusion

In conclusion, this study establishes the TyG-BMI as a robust predictor of adverse pregnancy outcomes in PCOS women undergoing their first FET. Elevated TyG-BMI demonstrated independent associations with increased miscarriage risk, reduced live birth rate, higher GDM incidence and LGA incidence. These findings highlight the critical role of pre-conception metabolic management in achieving reproductive success and safeguarding long-term maternal and offspring health. By incorporating TyG-BMI into routine clinical screening, physicians can identify high-risk PCOS patients who may benefit from targeted metabolic optimization prior to IVF, such as lifestyle interventions or pharmacologic therapies, to reduce the risk of adverse outcomes.

## Data Availability

The original contributions presented in the study are included in the article/**Supplementary Material**. Further inquiries can be directed to the corresponding authors.

## Glossary

PCOS: polycystic ovary syndrome
ART: assisted reproductive technologies
IVF: *in vitro* fertilization
GDM: gestational diabetes mellitus
LBW: low birthweight
LGA: large for gestational age
IR: insulin resistance
HOMA-IR: homeostatic model assessment for IR
BMI: body mass index
TyG: triglyceride glucose
ET: embryo transfer
OHSS: ovarian hyperstimulation syndrome
FET: frozen embryo transfer
ICSI: intracytoplasmic sperm injection
COS: controlled ovarian stimulation
GnRH: gonadotropin-releasing hormone
2PN: two pronuclei
FBG: fasting blood glucose
TC: total cholesterol
TG: triglycerides
LDL: low-density lipoprotein
HDL: high-density lipoprotein
AMH: anti-müllerian hormone
AFC: antral follicle account
HDP: hypertensive disorders of pregnancy
PTB: preterm birth
SGA: small for gestational age
SBP: systolic blood pressure
Gn: gonadotropin
OR: odds ratio
CI: confidence interval
RCS: restricted cubic spline
LASSO: least absolute shrinkage and selection operator
ROC: receiver operating characteristic
DBP: diastolic blood pressure
FSH: follicle-stimulating hormone
LH: luteinizing hormone
E2: estradiol
T: testosterone
PRL: prolactin
GnRH: gonadotropin-releasing hormone
AUC: area under the curve.
