# Supplementary material for: The triglyceride glucose-body mass index predicts adverse reproductive outcomes in women with polycystic ovary syndrome undergoing frozen embryo transfer
Source: Front Endocrinol (Lausanne). 2025 Jul 30;16:1629837. doi: 10.3389/fendo.2025.1629837 (PMC12343246; doi:10.3389/fendo.2025.1629837)
Supplement: Supplementary file 1 [file DataSheet1.docx]

Supplementary Material

# Supplementary Figures and Tables

## Supplementary Figures


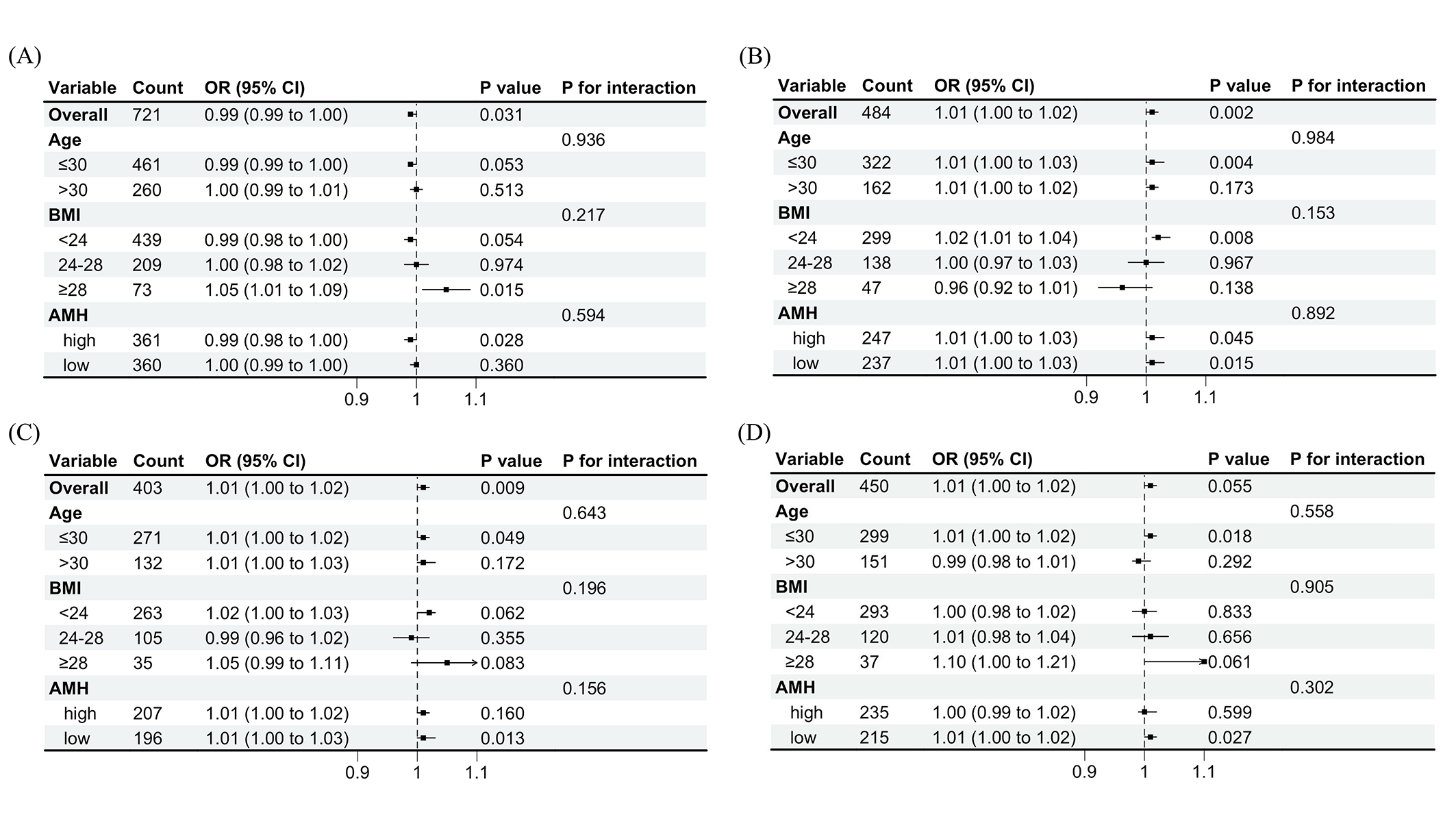


**Supplementary Figure 1.** Stratified analysis of the associations between TyG-BMI and reproductive outcomes. (A) Live birth rate. (B) Miscarriage rate. (C) GDM incidence. (D) LGA incidence.


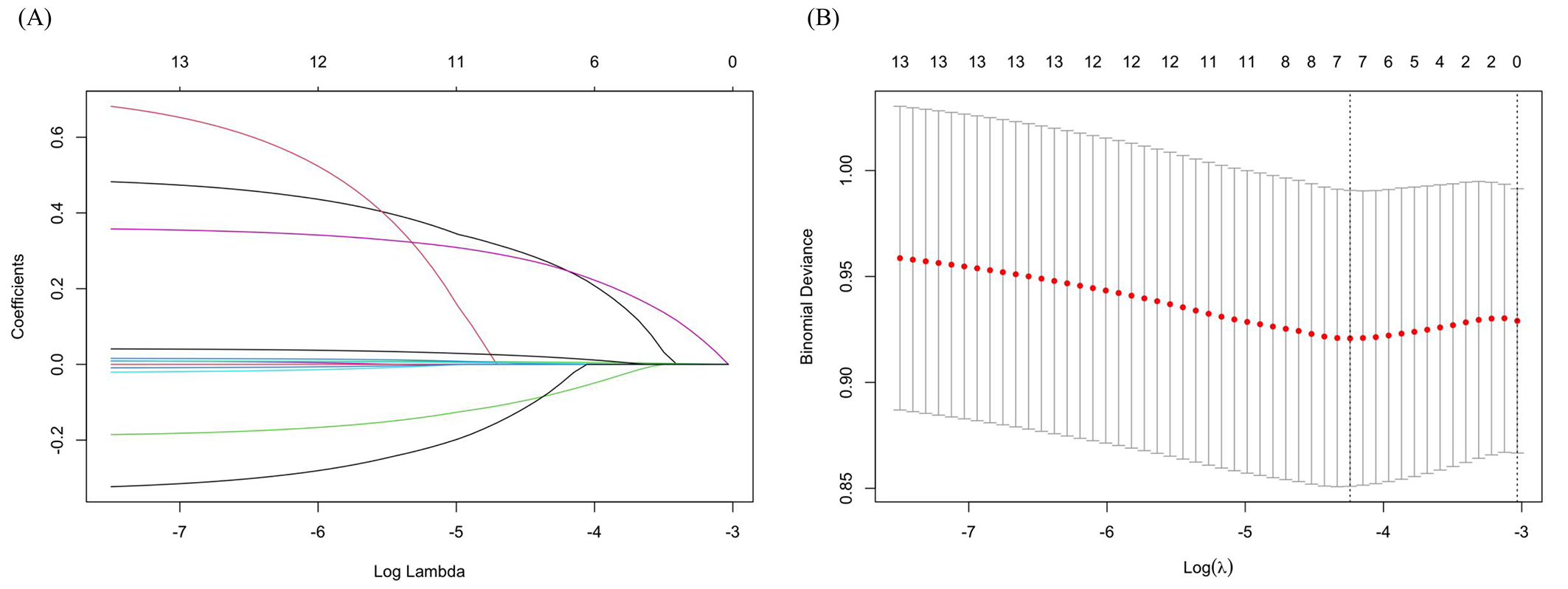


**Supplementary Figure 2.** (A) LASSO coefficient profiles of variables. Each continuous variable was shown as a colored line; (B) The lambda (λ) selection process in the LASSO regression. The optimal λ, with which zero variable using lambda.1se (right line) or seven variables using lambda.min (left line) with non-zero coefficients were screened out.

## Supplementary Tables

**Supplementary Table 1.** ROC analysis of different models for predicting miscarriage in PCOS women undergoing FET

| Models | AUC (95% CI) | Sensitivity | Specificity | Youden’ index | P value |
| --- | --- | --- | --- | --- | --- |
| TyG-BMI+Baseline | 0.667(0.603-0.730) | 0.853 | 0.441 | 0.294 | <0.001 |
| TyG-BMI | 0.627(0.564-0.690) | 0.843 | 0.380 | 0.223 | <0.001 |
| Baseline | 0.637(0.570-0.705) | 0.827 | 0.423 | 0.250 | <0.001 |

ROC, receiver operating characteristic; PCOS, polycystic ovary syndrome; FET, frozen embryo transfer; AUC, area under the curve; CI, confidence interval; TyG-BMI, triglyceride glucose-body mass index.

Baseline model included basal luteinizing hormone, basal follicle stimulating hormone, total cholesterol, testosterone, infertility type and controlled ovarian stimulation protocols.

**Supplementary Table 2.** ROC analysis of the full model for predicting miscarriage in subgroups

| Subgroups | AUC (95% CI) | Sensitivity | Specificity | Youden’ index | P value |
| --- | --- | --- | --- | --- | --- |
| BMI < 24kg/m^2^ | 0.743(0.666-0.820) | 0.778 | 0.637 | 0.415 | <0.001 |
| BMI ≥ 24kg/m^2^ | 0.662(0.565-0.759) | 0.949 | 0.325 | 0.274 | 0.001 |
| Age ≤ 30 | 0.702(0.625-0.780) | 0.766 | 0.594 | 0.360 | <0.001 |
| Age > 30 | 0.623(0.511-0.736) | 0.750 | 0.518 | 0.268 | 0.022 |
| low AMH | 0.673(0.573-0.772) | 0.703 | 0.596 | 0.299 | 0.001 |
| high AMH | 0.693(0.607-0.778) | 0.806 | 0.525 | 0.331 | <0.001 |

ROC, receiver operating characteristic; AUC, area under the curve; CI, confidence interval; BMI, body mass index; AMH, anti-müllerian hormone.

The full model included triglyceride glucose-body mass index, basal luteinizing hormone, basal follicle stimulating hormone, total cholesterol, testosterone, infertility type and controlled ovarian stimulation protocols.
